# Low Resistivity and High Carrier Concentration in SnO_2_ Thin Films: The Impact of Nitrogen–Hydrogen Annealing Treatments

**DOI:** 10.3390/nano15130986

**Published:** 2025-06-25

**Authors:** Qi-Zhen Chen, Zhi-Xuan Zhang, Wan-Qiang Fu, Jing-Ru Duan, Yu-Xin Yang, Chao-Nan Chen, Shui-Yang Lien

**Affiliations:** 1 Xiamen Key Laboratory of Development and Application for Advanced Semiconductor Coating Technology, The School of Opto-Electronic and Communication Engineering, Xiamen University of Technology, Xiamen 361024, China; 2022000023@xmut.edu.cn (Q.-Z.C.); 2222031241@stu.xmut.edu.cn (W.-Q.F.); 2422101008@stu.xmut.edu.cn (J.-R.D.); 2422101028@stu.xmut.edu.cn (Y.-X.Y.); 2 Institute of Optoelectronic Display, National & Local United Engineering Lab of Flat Panel Display Technology, Fuzhou University, Fuzhou 350002, China; 241116005@fzu.edu.cn; 3 School of Advanced Manufacturing, Fuzhou University, Quanzhou 362200, China; 4 Department of Computer Science and Information Engineering, Asia University, Taichung 413, Taiwan; chencn@asia.edu.tw

**Keywords:** PEALD, SnO_2_, annealing, ETL, PSCs

## Abstract

The tin dioxide (SnO_2_) thin films in this work were prepared by using plasma-enhanced atomic layer deposition (PEALD), and a systematic analysis was conducted to evaluate the influence of post-deposition annealing at various temperatures in a nitrogen–hydrogen mixed atmosphere on their surface morphology, optical behavior, and electrical performance. The SnO_2_ films were characterized by using X-ray diffraction (XRD), X-ray photoelectron spectroscopy (XPS), scanning electron microscopy (SEM), transmission electron microscopy (TEM), and Hall effect measurements. With increasing annealing temperatures, the SnO_2_ films exhibited enhanced crystallinity, a higher oxygen vacancy (O_V_) peak area ratio, and improved mobility and carrier concentration. These enhancements make the annealed SnO_2_ films highly suitable as electron transport layers (ETLs) in perovskite solar cells (PSCs), providing practical guidance for the design of high-performance PSCs.

## 1. Introduction

Perovskite solar cells (PSCs) have emerged as a highly promising class of photovoltaic technologies due to their remarkable power conversion efficiencies (PCEs), low fabrication costs, and compatibility with large-area substrates [1,2,3,4,5]. Within the device architecture, the electron transport layer (ETL) plays a pivotal role in facilitating efficient electron extraction, blocking holes, and influencing the overall performance and stability of the device. Tin dioxide (SnO_2_) is widely regarded as an ideal ETL candidate owing to its high electron mobility, excellent optical transparency, favorable energy band alignment with perovskite absorbers, and good chemical stability [6,7,8,9,10,11]. However, the electrical performance of SnO_2_ films is highly sensitive to their crystalline quality, oxygen vacancy (O_V_) concentration, and interface properties, all of which are strongly influenced by deposition technique and post-treatment conditions [12,13,14].

Recent advancements in light management strategies have introduced microstructured or patterned glass substrates in PSC architectures to enhance photon harvesting and reduce optical reflection [15,16,17,18]. These complex topographies demand deposition techniques capable of producing conformal, uniform, and pinhole-free films over uneven surfaces. Conventional deposition methods, such as sputtering or sol–gel processes, often struggle to meet these requirements due to limited step coverage and poor thickness control on textured substrates.

Compared with various physical vapor deposition methods, PEALD combines the advantages of atomic-level thickness control and excellent conformality inherent in ALD processes while enabling low-temperature deposition and broader material versatility through plasma activation [19,20,21,22,23,24]. These advantages make PEALD an ideal method for depositing SnO_2_ ETLs on microstructured glass substrates used in modern PSCs. However, the electrical and structural properties of PEALD-deposited SnO_2_ films can be further tuned through post-deposition thermal annealing, especially in a reducing atmosphere such as forming gas (N_2_/H_2_), which can modulate oxygen vacancy content and promote crystallization. Despite the promising application of PEALD-derived SnO_2_ in PSCs, comprehensive investigations into the influence of annealing temperature on the microstructural evolution and functional performance of these films remain limited [25].

In this work, SnO_2_ thin films were deposited by using PEALD, followed by post-deposition annealing at various temperatures in a N_2_/H_2_ (95:5) atmosphere. The influence of thermal treatment on the morphology, crystal structure, chemical composition, and electrical properties of the films was systematically examined through a combination of characterization techniques, including X-ray diffraction (XRD), X-ray photoelectron spectroscopy (XPS), scanning electron microscopy (SEM), transmission electron microscopy (TEM), and Hall effect measurements. The results provide insight into the thermal evolution of SnO_2_ films and demonstrate their suitability as ETLs for high-efficiency PSCs, especially in architectures employing microstructured glass substrates.

## 2. Materials and Methods

Silicon wafers and glass acted as substrates for the deposited SnO_2_ films after ultrasonic treatment with isopropanol ethanol and deionization for 10 min. Glass substrates (Corning**^®^** EXG, Corning, NY, USA, 100 mm × 100 mm × 0.2 mm) were used for electrical characterizations. Single-crystal p-type Si wafers (450 μm thickness, 50 Ω·cm) were used for structural characterizations including XRD, XPS, SEM, and TEM. The SnO_2_ films were deposited by using PEALD (R-200, Picosun, Espoo, Finland) by alternately exposing the sample to Tetrakis(dimethylamino)tin (TDMASn) and O_2_ plasma, in which TDMASn was employed as the tin precursor, while oxygen plasma served as the oxidant. The TDMASn precursor was maintained at 50 °C in a bubbler to ensure adequate vapor pressure, and high-purity argon (80 sccm) was used as the carrier and purge gas. The substrates were heated to 200 °C during the deposition process. Each ALD cycle consisted of a 1.6 s TDMASn pulse, followed by a 6 s Ar purge. Subsequently, O_2_ gas (150 sccm) was introduced and excited into plasma at a power of 2500 W, with an 11 s plasma exposure time and a 5 s purge step. A continuous TDMASn carrier gas flow of 120 sccm and a dilution flow of 400 sccm were maintained throughout the process to ensure stable precursor delivery. A total of 308 cycles were used to achieve a film thickness of approximately 41.26 nm. Including all precursor exposure and purge steps, the entire deposition process took around 2 h. The detailed process parameters are summarized in Table 1. SnO_2_ thin films were annealed at 300, 400, 500, and 600 °C using a vacuum tube furnace (YTGM 310-17, Shanghai Yetuo Technology Co., Shanghai, China). To prevent thermal deformation of the glass substrate, whose softening point is approximately 700 °C, the annealing temperature in this study was restricted to a maximum of 600 °C [26,27,28]. Prior to heating, the furnace tube was evacuated to a base pressure of 1.5 mTorr (0.2 Pa) to minimize contamination. The samples were loaded into alumina (Al_2_O_3_) crucibles and subjected to a controlled heating process at a rate of 5 °C/min until the target annealing temperature was reached. They were then maintained at this temperature for 1 hour (isothermal hold), followed by natural cooling to below 100 °C. A forming gas atmosphere (N_2_/H_2_ = 95:5) was introduced at the beginning of the heating process and maintained throughout the entire annealing cycle. A chamber pressure of 15 mTorr was sustained during the ramping period and increased to 1.5 Torr during the one-hour annealing stage, while the gas flow rate remained fixed at 3.0 L/min.

The crystalline structure of the SnO_2_ thin films was characterized via grazing incidence X-ray diffraction (GIXRD; Rigaku TTRAX III, Ibaraki, Japan) using Cu Kα radiation (λ = 0.15418 nm) at a fixed incident angle of 0.5°, which effectively suppresses substrate signals and enhances the diffraction signal from the thin films. The elemental composition and chemical states were analyzed via X-ray photoelectron spectroscopy (XPS; ESCALAB 250Xi, Thermo Fisher Scientific, Waltham, MA, USA). Al Kα radiation (1486.6 eV) was used as the X-ray source, which typically provides an information depth of approximately 5–10 nm, depending on the material and detection angle. To minimize the effect of surface contamination and obtain representative chemical information from the near-surface region, Ar^+^ ion sputtering was performed for 30 seconds at a rate of ~0.275 nm/s prior to data acquisition, effectively removing approximately 8–9 nm from the film surface. As a result, the obtained XPS spectra reflect the sub-surface composition rather than just the outermost surface, ensuring more reliable chemical state analysis. Electrical properties of the SnO_2_ thin films, including resistivity, carrier concentration, and mobility, were measured using a Hall effect measurement system (HMS-5000; Ecopia, Anyang, Republic of Korea). Hall measurements were performed in the Van der Pauw configuration at room temperature (~25 °C). To eliminate parasitic conduction, the SnO_2_ films were deposited on insulating glass substrates. Square-shaped samples with dimensions of approximately 1 cm × 1 cm were prepared, and four small indium contacts were manually pressed onto the corners to ensure good ohmic contact. The surface morphology of the films was examined using field emission scanning electron microscopy (FESEM; Sigma 500, Zeiss, Oberkochen, Germany), while cross-sectional microstructures were observed via transmission electron microscopy (TEM; Talos F200X, Thermo Fisher Scientific, Hillsboro, OR, USA).

## 3. Results and Discussion

**Figure 1:**
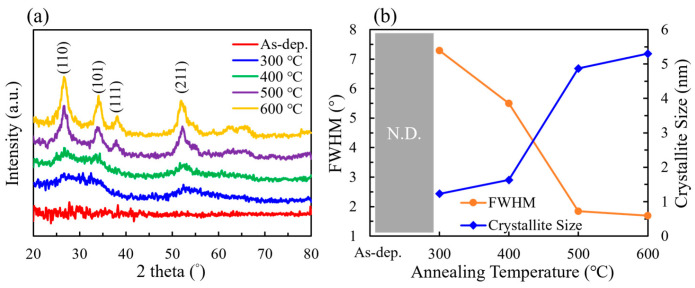
(**a**) XRD patterns and (**b**) FWHM and crystallite size of SnO_2_ films at different annealing temperatures.

The grazing incidence X-ray diffraction (GIXRD) patterns of SnO_2_ films subjected to annealing at various temperatures are shown in Figure 1a and were analyzed to investigate their crystalline structure. The XRD analysis revealed that higher annealing temperatures promoted crystallization of the SnO_2_ films, evidenced by the emergence and sharpening of characteristic diffraction peaks. The intensity of these peaks increased with higher crystallinity. Diffraction peaks were observed at 2θ = 26.98°, 31.14°, 38.18°, and 52.06°, corresponding to the (110), (101), (111), and (211) planes of SnO_2_, respectively, as indexed in the standard JCPDS database (No. 06-0318). Additionally, with increasing annealing temperature, the full width at half maximum (FWHM) of the diffraction peaks decreased from 7.29° at 300 °C to 1.69° at 600 °C, as shown in Figure 1b, indicating an increase in grain size. Scherrer equation calculations revealed that the grain size grew from 1.23 nm at 300 °C to 5.30 nm at 600 °C [29,30]. The peak positions remained stable across all annealing conditions, suggesting that no significant changes occurred in the lattice parameters. Furthermore, the diffraction peaks became sharper and more symmetrical, indicating a more well-ordered crystal structure with fewer defects.

**Figure 2:**
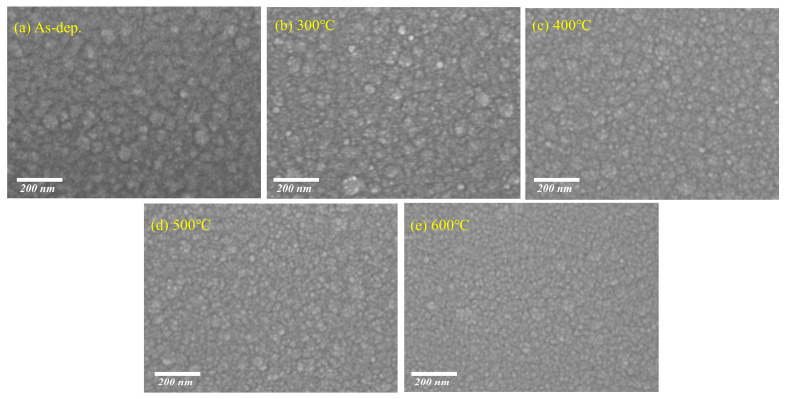
Topographical SEM images of SnO_2_ thin films at different annealing temperatures: (**a**) as-deposited SnO_2_ film, (**b**) SnO_2_ film annealed at 300 °C, (**c**) SnO_2_ film annealed at 400 °C (**d**) SnO_2_ film annealed at 500 °C, and (**e**) SnO_2_ film annealed at 600 °C.

Figure 2a–e show the surface morphology of SnO_2_ thin films annealed at different temperatures, as observed by top-view SEM (scale bar: 200 nm). The films were deposited on Si substrates. At lower annealing temperatures, the surface exhibits notable agglomeration, where small nanocrystalline domains tend to cluster into larger, irregularly shaped aggregates. With increasing annealing temperature, these aggregates are significantly reduced, and the surface becomes smoother and more uniform, indicating enhanced film densification and improved morphological uniformity [31,32,33]. Although no distinct crystalline grains are visible in the SEM images, this morphological evolution is consistent with the XRD results, which show a gradual decrease in the FWHM of diffraction peaks—suggesting enhanced crystallinity and grain growth. These findings imply that N_2_/H_2_ annealing not only reduces surface agglomeration but also promotes structural ordering at the nanoscale.

**Figure 3:**
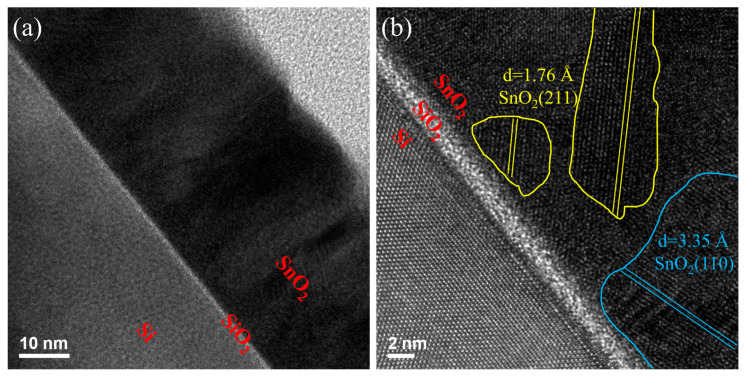
Cross-sectional transmission electron microscopy (TEM) images of the Si/buffer/SnO_2_ multilayer structure annealed at 600 °C in a N_2_/H_2_ atmosphere: (**a**) low magnification and (**b**) high magnification.

To evaluate the microstructural characteristics of the SnO_2_ thin films annealed at 600 °C, cross-sectional TEM was performed at magnifications of 300 K and 800 K. The samples consist of a Si substrate with an interfacial SiO_2_ layer of approximately 2 nm, serving as a buffer between the substrate and the SnO_2_ film. At low magnification (300 K), as shown in Figure 3a, the overall film structure is clearly observed, with a uniform SnO_2_ layer atop the Si/SiO_2_ interface. The SiO_2_ interlayer appears as a continuous, amorphous contrast layer between the crystalline Si substrate and the polycrystalline SnO_2_ film. Figure 3b presents high-resolution TEM (800 K magnification) images showing well-ordered lattice fringes in the SnO_2_ film, further evidencing the improved crystallinity achieved through thermal treatment. The measured lattice spacings are approximately 0.334 nm and 0.263 nm, which correspond to the (110) and (211) crystal planes of tetragonal rutile SnO_2_ (JCPDS No. 06-0318), respectively. These planes are clearly marked in the image, confirming the oriented grain growth and crystalline nature of the film. The presence of sharp interfaces and well-ordered lattice structures suggests that annealing at 600 °C effectively promotes SnO_2_ crystallization while preserving the integrity of the interfacial SiO_2_ layer. Such structural improvements are expected to enhance carrier mobility and are consistent with the observed electrical performance improvements.

**Figure 4:**
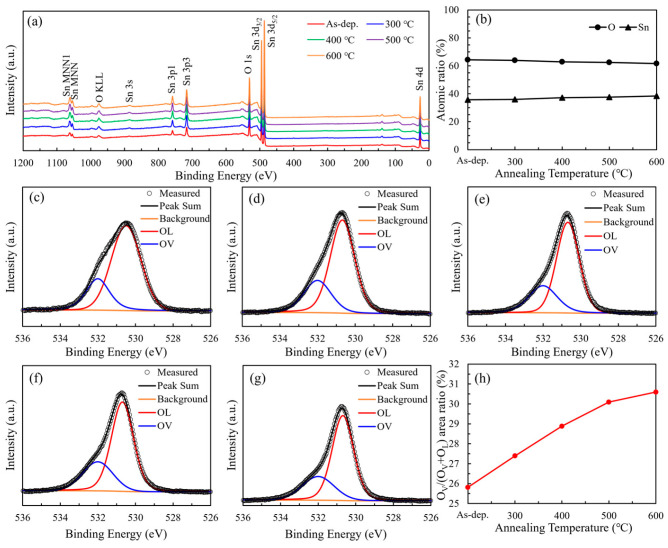
(**a**) XPS survey spectra of SnO_2_ films; (**b**) atomic ratio of Sn to O at different annealing temperatures; high-resolution O 1s spectra of (**c**) as-deposited SnO_2_ film; (**d**) SnO_2_ film annealed at 300 °C; (**e**) SnO_2_ film annealed at 400 °C; (**f**) SnO_2_ film annealed at 500 °C; (**g**) SnO_2_ film annealed at 600 °C; and (**h**) the O_V_ peak area ratio of the SnO_2_ films at different annealing temperatures.

The X-ray photoelectron spectroscopy (XPS) survey spectra of SnO_2_ films annealed at different temperatures are given in Figure 4a, which exhibit distinct peaks corresponding to Sn and O, with no detectable additional impurity peaks, indicating high sample preparation purity and no introduction of unexpected impurities. Figure 4b illustrates the variation in atomic concentrations of Sn and O in the SnO_2_ films as a function of annealing temperature. As the temperature increases, the atomic percentage of Sn exhibits a gradual rise, whereas that of O shows a corresponding decline. Consequently, the O/Sn atomic ratio decreases from 1.80 at 300 °C to 1.61 at 600 °C. This trend suggests a progressive loss of oxygen, which is indicative of the formation of oxygen vacancies. Figure 4c–g display the high-resolution O 1s XPS spectra of SnO_2_ thin films subjected to annealing at various temperatures. The broad peak profiles indicate the presence of multiple oxygen-related chemical states. Deconvolution of the O 1s spectra reveals two distinct components: a peak at 530.1 eV corresponding to lattice oxygen (O_L_), and another at 531.1 eV associated with oxygen vacancies (O_V_). As illustrated in Figure 4 h, the area ratio of O_V_ to the total oxygen content [O_V_/(O_V_ + O_L_)] increases with rising annealing temperature. Specifically, the O_V_/(O_V_ + O_L_) ratio of the as-deposited SnO_2_ film is 25.81%, and this value gradually rises to 30.59% with increasing annealing temperature from 300 °C to 600 °C, indicating an enhancement in oxygen vacancy concentration induced by thermal treatment. This trend indicates that hydrogen in the N_2_/H_2_ (95:5) annealing atmosphere acts as a reducing agent, reacting with lattice oxygen to generate H_2_O and oxygen vacancies. This mechanism is further supported by the observed decrease in overall oxygen content (Figure 4b), confirming that high-temperature annealing under reducing conditions facilitates the formation of oxygen vacancies in SnO_2_ thin films.

**Figure 5:**
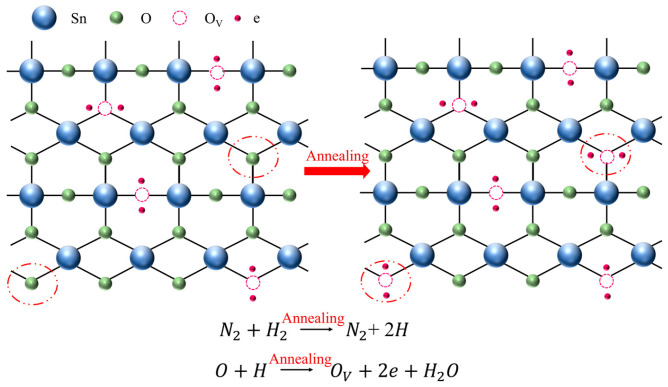
A schematic mechanism of annealing-induced properties.

To further elucidate the origin of oxygen vacancies (O_Vs_) in the SnO_2_ films annealed under a reducing atmosphere, a schematic diagram is presented in Figure 5. During annealing in a nitrogen–hydrogen (N_2_/H_2_) ambient atmosphere, hydrogen molecules diffuse to the film surface and react with lattice oxygen atoms (O_L_) in the SnO_2_ crystal. This reaction forms gaseous H_2_O, which desorbs from the lattice, thereby removing lattice oxygen and generating oxygen vacancies (O_VS_). The creation of oxygen vacancies releases free electrons, which act as shallow donors and contribute to the increased carrier concentration. The overall reaction can be expressed as follows:

O_L_ (lattice oxygen) + H_2_ → H_2_O (gas) + O_V_ (oxygen vacancy)

This mechanism aligns with the XPS results shown in Figure 4b,h, where a clear reduction in the O/Sn atomic ratio and an increase in the O_V_/(O_V_ + O_L_) peak area ratio are observed with increasing annealing temperature.

**Figure 6:**
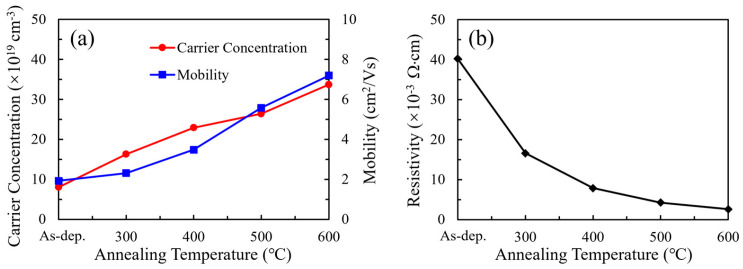
(**a**) Carrier concentration, mobility, and (**b**) resistivity of SnO_2_ thin films at different annealing temperatures.

Hall effect measurements were employed to investigate the electrical characteristics of the SnO_2_ thin films, with the results summarized in Figure 6. To ensure accurate electrical characterization, Hall measurements were performed on SnO_2_ films deposited on insulating glass substrates, thereby eliminating any electrical contribution from the substrate. The thickness values used to calculate the carrier concentration were determined using spectroscopic ellipsometry. For the as-deposited film, the initial thickness was approximately 41.26 nm. Since annealing could cause mild surface etching, the film thickness was remeasured after each annealing condition. The updated thickness values—39.89 nm (300 °C), 40.53 nm (400 °C), 41.56 nm (500 °C), and 39.31 nm (600 °C)—were used in the Hall analysis to ensure accurate estimation of carrier concentrations. This analysis provides insight into the variations in resistivity, carrier concentration, and mobility as a function of annealing temperature. The carrier concentration exhibited a significant increase with rising annealing temperature, rising from 8.05 × 10^19^ to 3.37 × 10^20^ cm^−3^. This enhancement is attributed to the elevated concentration of oxygen vacancies, as confirmed by XPS analysis. These vacancies act as shallow donors, supplying free electrons to the conduction band and thereby improving n-type conductivity. The Hall mobility also exhibits a positive correlation with annealing temperature, increasing from 1.93 to 7.19 cm^2^/V·s. This improvement is likely associated with grain growth, enhanced crystallinity, and reduced carrier scattering. Correspondingly, the resistivity, depicted in Figure 6b, shows a pronounced reduction from 4.02 × 10^−2^ Ω·cm to 2.58 × 10^−3^ Ω·cm as the annealing temperature increases. These improvements in electrical performance are particularly beneficial for SnO_2_ when employed as ETLs in PSCs. Higher carrier concentration and mobility facilitate more efficient electron extraction and suppress charge recombination at the interface, thereby contributing to improved fill factors and overall device efficiency.

To explore the influence of different gas environments on the electrical properties of SnO_2_ thin films, annealing was carried out in six different atmospheres at 600 °C: pure argon, forming gas (N_2_:H_2_ = 95%:5%), vacuum, oxygen, ozone, and air. The purpose was to compare the effects of reducing and oxidizing environments. The results, shown in Table 2, demonstrate that reducing atmospheres significantly increased the carrier concentration, whereas oxidizing conditions tended to reduce carrier concentration but improve mobility. Notably, N_2_:H_2_ annealing yielded the highest carrier concentration (3.37 × 10^20^ cm^−3^) and lowest resistivity (2.58 × 10^−3^ Ω·cm), indicating enhanced electrical performance under moderately reducing conditions. It provides a comprehensive insight into gas-environment-dependent tuning of SnO_2_ films and validates the potential of forming gas annealing for optimizing electron transport layers in transparent conductive oxide (TCO) applications.

To further validate the electrical advantages of the SnO_2_ films developed in this work, a comparative analysis was performed against previously reported films fabricated by using various deposition methods and subjected to post-annealing, as summarized in Table 3 [34,35,36,37,38]. The SnO_2_ films prepared via PEALD and annealed at 600 °C in a N_2_/H_2_ atmosphere exhibit a significantly lower resistivity (2.58 × 10^−3^ Ω·cm) and a much higher carrier concentration (7.19 × 10^20^ cm^−3^) than most reported counterparts. This superior conductivity is primarily attributed to the increased concentration of oxygen vacancies induced by high-temperature annealing in a reducing ambient atmosphere, as confirmed by XPS analysis. Although the Hall mobility (7.19 cm^2^/V·s) is relatively lower compared to films deposited by sputtering, pulsed laser deposition (PLD), sol–gel processes, and thermal ALD, the overall electrical performance—particularly the ultra-low resistivity and high carrier density—demonstrates the effectiveness of the PEALD approach in optimizing film conductivity. These results suggest that PEALD-derived SnO_2_ films, with their excellent charge transport properties and interface quality, hold strong potential as ETLs in PSCs and other advanced optoelectronic devices.

## 4. Conclusions

This study demonstrates that PEALD-grown SnO_2_ thin films, when subjected to post-annealing in a N_2_/H_2_ atmosphere, exhibit significantly enhanced structural and electrical properties. With increasing annealing temperature, the films transitioned from amorphous to crystalline phases, showing improved grain size, crystallinity, and surface uniformity. XPS analysis revealed an increase in oxygen vacancies, which acted as shallow donors and contributed to a substantial rise in carrier concentration. Hall measurements confirmed that resistivity decreased to 2.58 × 10^−3^ Ω·cm and carrier concentration increased to 3.37 × 10^20^ cm^−3^. Overall, the results highlight that PEALD combined with reducing-atmosphere annealing is an effective strategy for engineering high-quality SnO_2_ thin films, offering great potential for use as ETLs in PSCs and other advanced optoelectronic devices.

## Figures and Tables

**Table 1:** Preparation parameters of PEALD-SnO_2_ thin films.

Parameter	Value
Bubbler temperature (°C)	50
Substrate temperature (°C)	200
TDMASn pulse time (s)	1.6
TDMASn purge time (s)	6
O_2_ pulse time (s)	11
O_2_ purge time (s)	5
Ar flow rate (sccm)	80
O_2_ flow rate (sccm)	150
O_2_ plasma power (W)	2500
TDMASn carry gas flow rate (sccm)	120
TDMASn dilute gas flow rate (sccm)	400
Post-annealing temperature (°C)	300–600
Post-annealing gas	N_2_ (95%) + H_2_ (5%)
Post-annealing gas flow rate (L/min)	3

**Table 2:** Electrical properties of SnO_2_ films annealed in different atmospheres at 600 °C.

Annealing Atmosphere	Carrier Concentration (cm^−3^)	Mobility (cm^2^/V·s)	Resistivity (Ω·cm)
N_2_:H_2_	3.37 × 10^20^	7.19	2.58 × 10^−3^
Argon	1.03 × 10^20^	6.53	9.25 × 10^−3^
Vacuum	2.58 × 10^20^	3.22	7.52 × 10^−3^
Oxygen	3.15 × 10^19^	15.23	1.30 × 10^−2^
Ozone	5.78 × 10^18^	19.46	5.60 × 10^−2^
Air	1.03 × 10^19^	9.41	6.46 × 10^−2^

**Table 3:** Comparison of electrical properties of SnO_2_ films prepared via different deposition methods after annealing.

Deposition Method	Annealing Conditions	R (Ω·cm)	n (cm^−3^)	Mobility (cm^2^/V·s)	T (nm)	Ref.
sol–gel	Air, 700 °C, 2 h	72.67	6.25 × 10^15^	13.7	30.8	[34]
PLD	O_2_, 600 °C	1.97 × 10^–2^	3 × 10^18^	106	700	[35]
MS	None (600 °C substrate heating)	1.75 × 10^−3^	1.6 × 10^20^	22.48	300	[37]
DC	None (150 °C substrate heating)	4.5 × 10^−3^	8.9 × 10^19^	20.77	12.5	[38]
thermal ALD	N_2_, 400 °C, 1 h	~8.8 × 10^–3^	3.3 × 10^19^	21.55	20	[36]
PEALD	N_2_/H_2_ (95:5), 600 °C, 1 h,	2.58 × 10^–3^	3.37 × 10^20^	7.19	40	This work

Ref. = reference; n = carrier concentration; R = resistivity; MS = magnetron sputtering; DC = DC sputtering; T = thickness.

## Data Availability

The data are contained within the article.
